# Factors associated with internet use and health information technology use among older people with multi-morbidity in the United States: findings from the National Health Interview Survey 2018

**DOI:** 10.1186/s12877-022-03410-y

**Published:** 2022-09-06

**Authors:** Wenbo He, Liujiao Cao, Rui Liu, Yi Wu, Wei Zhang

**Affiliations:** 1 Institute of Hospital Management, West China Hospital of Sichuan University, Chengdu, Sichuan China; 2 Saw Swee Hock School of Public Health, National University of Singapore, Singapore, Singapore; 3 West China School of Nursing/West China Hospital, Sichuan University, Chengdu, Sichuan China; 4 Department of Rehabilitation Medicine Center, West China Hospital, Sichuan University, Chengdu, Sichuan China; 5 Key Laboratory of Rehabilitation Medicine in Sichuan Province, Chengdu, Sichuan China; 6 Department of Epidemiology, School of Public Health, Harbin Medical University, Harbin, Heilongjiang, China; 7 West China Biomedical Big Data Center, West China Hospital of Sichuan University, Chengdu, Sichuan China

**Keywords:** Internet use, HIT, Physical multi-morbidity, Older adults

## Abstract

**Background:**

The number of older adults with physical multi-morbidity is increasing. As Internet-based eHealth and mHealth increasingly require patients to use technology, it is important to examine the use of Internet/health information technology (HIT) among older adults with physical multi-morbidity. Here we examine the distribution of physical multi-morbidity, Internet use, and HIT use, and further explored the factors associated with Internet use and HIT use among older adults with physical multi-morbidity.

**Methods:**

One wave of data from the 2018 US National Health Interview Survey (NHIS) was analysed. We included respondents aged 65 years and older. We used 13 physical non-communicable diseases to measure physical multi-morbidity. Descriptive statistics and logistic regression models, with sociodemographic factors, health status, health insurance, health care service use, and satisfaction with health care as covariates, were used to examine the research questions.

**Results:**

Of 72,746 respondents in NHIS, 7060 were eligible for our analysis. 5380 (76.2%) eligible respondents had physical multi-morbidity in this study. Overall, 60% of older adults reported using the Internet, with 38.9% using eHealth services (defined as looking up health information online, filling a prescription, scheduling an appointment with a health care provider, or communicating with a health care provider via email). Gender, age, marital status, region, race, education, and family income were significant factors associated with the Internet and HIT use among people with multi-morbidity. The study also showed that after adjusting for confounders, good health status, having Medicare, receiving home care from a health professional, and low satisfaction with health care were positive predictors of the Internet and HIT use.

**Conclusions:**

In summary, our study found that Internet and HIT use among older patients with chronic diseases is far from the Healthy People 2030 target. Internet and HIT use vary depending on a number of sociodemographic factors. Relevant influencing factors should be fully considered in health education interventions promoted.

## Background

The recognition that chronic diseases are a major challenge to global health is undisputed [1, 2]. In its 2021 report on the global chronic disease challenge, WHO pointed out that non-communicable diseases cause 41 million deaths per year, equivalent to 71% of all global deaths [3]. More seriously, physical multi-morbidity (the co-occurrence of two or more chronic diseases) is also on the rise worldwide [4]. People with multi-morbidity often have poorer health outcomes, such as increased mortality, disability, decreased physical function, and reduced quality of life [5].

In the USA, chronic diseases are the leading cause of poor health, disability, and death, and account for the majority of medical expenditures. About half (50.9%) of adults have at least one chronic disease, and 26% have two or more diseases [6]. On the one hand, the older adults in the United States are facing a persistently high prevalence of risk factors including the increasingly sedentary, unhealthy lifestyle and other behaviors, and on the other hand, they are faced with an increase in more ill problems caused by an increase in life expectancy [7, 8]. The large number and high incidence of chronic diseases have brought huge challenges and costs to public health and health care systems [8], so how to carry on scientific intervention to chronic disease is particularly important.

Adequate management of chronic diseases has proven to be resource-intensive and requires coordinated and dedicated efforts from teams of patients and healthcare providers [9, 10]. In recent years, technological advances, such as mHealth and eHealth are bringing about mechanisms for effective and affordable health care delivery and education [11]. Healthy People 2030 highlights the desire to increase the use of patient portals, particularly the proportion of persons who use health information technology (HIT) to track health care data or communicate with providers, and the proportion of persons who have access to quality (reliable and easy to use) digital health tools and information [12]. The Internet is and will continue to be a major source for people to receive health information as it plays an increasingly important role in health care and as individuals become more aware and receptive to new technologies such as the Internet [13]. Studies have shown that greater participation in the Internet and social media may be related to the improvement of health behaviors and health conditions, indicating the growing important of the Internet in health maintenance [14].

The Internet has been recognized as an increasingly popular channel for doctor's access, with more Americans using it to search for health information and perform other health-related activities, such as remotely filling prescriptions and using E-mail to communicate with their healthcare provider [15, 16]. This new form of communication provides broader and open access to information and advices on how to maintain or improve health and manage illness. The use of the Internet and health information may be particularly beneficial to older adults seeking health information [17, 18]. However, little is known about the proportion of older adults with physical multi-morbidity who use the Internet to search for health information and communicate with health care providers online [11, 19]. In addition, less attention has been paid to how HIT is used in older with physical multi-morbidity compares to US Healthy People 2030 objectives. In fact, this information is necessary because people with physical multi-morbidity often have complex management needs and potentially face barriers to HIT, but may benefit greatly from eHealth services [11].

In addition, exploring the association between sociodemographic factors and the Internet and HIT use by people with physical multi-morbidity may provide new insights to better promote this technological approach to care. It is well-known that patients who are older, of lower socioeconomic status, or with lower levels of education are less likely to engage in eHealth activities [19]. Studies have shown that compared with non-older groups, older patients are more dependent on their healthcare providers for information, rather than relying solely or heavily on the Internet for health information (and may not consider the Internet to be a credible, reliable, or easy to navigate resource) [20]. It is therefore important to understand the sociodemographic factors that influence the Internet and HIT use in patients with physical multi-morbidity to assist vulnerable populations and advance the progress of health equity.

Although the Internet is expected to become an additional complementary resource for current and future diseased groups, systematic analysis of the Internet usage patterns among the older adults, especially for patients with physical multi-morbidity, is still lacking. In response to these gaps in the literature, the current study described Internet and HIT use in older adults with physical multi-morbidity (objective 1). We further identified a range of factors associated with Internet and HIT use in older adults with physical multi-morbidity (objective 2).

## Methods

### Data and sample

Data from the 2018 US National Health Interview Survey (NHIS) public-use data files were downloaded from the CDC’s National Center for Health Statistics website, which contains a variety of health information Internet use [21]. As an annual, nationally representative, cross-sectional household survey, NHIS provides information on the health and health care access of the civilian, non-institutionalized US population [22]. The NHIS obtains data through a complex multistage sample design that involves the stratification and clustering of specific population subgroups. For each sampled family, a face-to-face interview is conducted with an adult family member, who answers questions about the demographic and health conditions of each family member. A detailed description of the objectives and methods of NHIS has been reported elsewhere [23]. Since these data are provided to the public in an identifiable format, this study was exempt from review by the Institutional Review Board. In the 2018 NHIS annual data, a sample of 72,746 adult respondents between the ages 18 and 85 was obtained (to protect confidentiality among the oldest adults, all age variables were top-coded to “85 years and older” (85 +) in the HNIS database). In this study, we focused on patients aged 65 years and older with physical multi-morbidity, and we excluded respondents who had missing values of dependent or independent variables.

### Measures

#### Physical multi-morbidity

We defined multi-morbidity as the presence of two or more physical chronic non-communicable diseases [22]. We used 13 non-communicable diseases to measure physical multi-morbidity, including diagnosed hypertension, high cholesterol, coronary heart disease, angina pectoris, heart condition, stroke, chronic obstructive pulmonary disease, asthma, cancer, diabetes, failing kidneys, liver condition, and arthritis. We counted the number of non-communicable diseases for each participant to identify those who had physical multi-morbidity.

#### Internet use

Respondents were asked if they had used the Internet in the past year (Yes = 1 and No = 0).

#### Health information technology use

Respondents were asked if they had (1) looked up health information on the Internet, (2) filled a prescription on the Internet, (3) scheduled a medical appointment on the Internet, and (4) communicated with a health care provider by email in the past 12 months. In this study, HIT use refers to any of these 4 aspects.

#### Health status

In NHIS, self-rated health was obtained by asking respondents, “Would you say your health, in general, is excellent, very good, good, fair, or poor?” We assigned "excellent", "very good" and “good” to 1, "fair" to 2, and "poor" to 3.

#### Health insurance

Respondents were asked if they had Medicare, Medicaid, private health insurance, and veterans/military insurance coverage in the past 12 months (yes = 1 and no = 0 for each).

#### Health care use

Respondents were asked if they (1) saw/talked to a medical specialist, (2) saw/talked to a general doctor, (3) received home care from a health professional, and (4) received health care 10 or more times in the past 12 months (yes = 1 and no = 0 for each).

#### Sociodemographic factors

A set of sociodemographic factors were included: (1) gender, (2) age (65—74, 75—84, and ≥ 85 years), (3) marital Status (Currently married, Widowed, Divorced/separated, Never married), (4) region (Northeast, Midwest, South, West), (5) race (Hispanic, Non-Hispanic White, Non-Hispanic Black, Non-Hispanic Asian, Non-Hispanic All other race groups), (6) highest education (High school and below, Some college, College, and Master degree and above), (7) the percentage of family income to the federal poverty guidelines (FPG) (< 200% FPG, 200%-399% FPG, ≥ 400% FPG).

### Data analysis

Statistical analyses were performed using the SPSS statistics 22.0 (IBM Corp. Released 2013. IBM SPSS Statistics for Windows, Version 22.0. Armonk, NY: IBM Corp). Some covariates contained missing values, and the proportion of missing values was less than 5% [22]. Thus, we replaced the missing data with the mean of their integrity items. Frequency and percentages were calculated to describe sociodemographic parameters and level distributions among respondents. We used logistic regression models with Internet and HIT use as dependent variables to determine factors that affect use of Internet and HIT among older adults with multi-morbidity. We report associations as unadjusted rates (uORs) and odds ratios adjusted (aORs) for gender, age, marital status, region, race, highest education, family income, health status, health insurance, and health care use. A significance level of 95% was used with α = 0.05.

## Results

Table 1 presents the socio-demographic characteristics of all respondents (*n* = 7060) and who with Internet use (*n* = 4061), HIT use (*n* = 3030), and multi-morbidity (*n* = 5380). The median age of all respondents was 74 years (IQR 65–85) in 2018. 2966 (42.1%) respondents were male, 4094 (57.9%) were female, and 3261 (46.2%) were currently married. About one-third (37.1%) respondents were living in the south area in the US, 5468 (77.5%) of them were non-hispanic white, 3017 (42.7%) respondents had high school education or below, and 2728 (38.6%) respondents had family incomes as a percentage of FPG of 200%—399%. More than 70% (5551,78.6%) of respondents reported good health, have health insurance (Medicare) (6284, 89.0%), and 6090 (86.3%) reported seeing/talking to a general doctor in the past 12 months. In addition, the proportion of older adults with physical multi-morbidity increased substantially with age. In 2018, the overall prevalence of physical multi-morbidity was 76.2%, with 3016 respondents aged 65–74 years having physical multi-morbidity and 656 older adults aged 85 years and above having physical multi-morbidity.

**Table 1:** Socio-demographic characteristics of all respondents and who with Internet use, HIT use, and multi-morbidity in 2018

Characteristics	**All respondents (*n* = 7060)**		**Internet use(*n* = 4061)**		**HIT use(*n* = 3030)**		**Multi-morbidity(*n* = 5380)**	
	N	%	N	%	N	%	N	%
**Gender**								
Male	2966	42.1	1758	43.3	1287	42.5	2278	42.3
Female	4094	57.9	2303	56.7	1743	57.5	3102	57.7
**Age**								
65–74	4118	58.3	2809	69.2	2157	52.4	3016	56.1
75–84	2116	30	1037	25.5	743	24.5	1708	31.7
85 +	826	11.7	215	5.3	130	4.3	656	12.2
**Marital Status**								
Currently married	3261	46.2	2167	53.4	1669	55.1	2419	45
Widowed	2015	28.5	835	20.6	580	19.1	1625	30.2
Divorced/separated	1329	18.8	800	19.7	591	19.5	1006	18.7
Never married	455	6.4	259	6.4	190	6.3	330	6.1
**Region**								
Northeast	1253	17.7	716	17.6	565	18.6	954	17.7
Midwest	1640	23.2	950	23.4	678	22.4	1278	23.8
South	2616	37.1	1430	35.2	1060	35	2016	37.5
West	1551	22	965	23.8	727	24	1132	21
**Race**								
Hispanic	516	7.3	165	4.1	115	3.8	378	7
Non-Hispanic White	5468	77.5	3445	84.8	2596	85.7	4150	77.1
Non-Hispanic Black	731	10.4	283	7	198	6.5	589	10.9
Non-Hispanic Asian	271	3.8	139	3.4	99	3.3	198	3.7
Non-Hispanic All other race group	74	1	29	0.7	22	0.7	65	1.2
**Highest education**								
High school and below	3017	42.7	1017	25	653	21.6	2293	44.5
Some college	1237	17.5	835	20.6	621	20.5	969	18
College	743	10.5	488	12	388	12.8	563	10.5
Master degree and above	2063	29.2	1721	42.4	1368	45.1	1455	27
**Family income to the FPG**^**a**^								
< 200% FPG	2128	30.1	749	18.4	516	17	1681	31.2
200%-399% FPG	2728	38.6	1567	38.6	1145	37.8	2089	38.9
≥ 400% FPG	2204	31.2	1745	43	1369	45.2	1610	29.9
**Health status**								
Good	5551	78.6	3511	86.5	2599	85.8	4012	74.6
Fair	1143	16.2	443	10.9	346	11.4	1021	19
Poor	366	5.2	107	2.6	85	2.8	347	6.4
**Health insurance**								
Private health insurance	3156	44.7	2056	50.6	1536	50.72	2408	44.8
Medicare	6284	89	3602	88.7	2696	89	4843	90
Medicaid	562	8	138	3.4	100	3.3	4469	8.7
Military health care	611	8.7	340	8.4	258	8.5	507	9.4
**Health care use in the past 12 months**								
Seen/talked to a medical specialist	3370	47.7	2062	50.8	1706	56.3	2878	53.5
Seen/talked to a general doctor	6090	86.3	3541	87.2	2671	88.2	4833	89.8
Received home care from health professional	646	9.2	228	5.6	189	6.2	581	10.8
receive care 10 + times	1420	20.1	742	18.3	631	20.8	1292	24
**Satisfied with health care**								
Very satisfied	5337	75.6	3135	77.2	2295	75.7	4005	74.4
Somewhat satisfied	1441	20.4	781	19.2	617	20.4	1138	21.2
Somewhat dissatisfied	201	2.8	106	2.6	88	2.9	167	3.1
Very dissatisfied	81	1.1	39	1	30	1	70	1.3

^a^ *FPG* federal poverty guidelines

As shown in Table 2, in general, approximately 60.0% of the respondents used the Internet. Among those who used the Internet, the majority (45.9%) used it with a frequency of every day, and only 1% (74) of users with a frequency of once a month. Although Internet users’ most common HIT use was for health information seeking (38.9%), between 9.2% and 13.1% also engaging in the other 3 types of HIT use. In addition, about 59.1% of people with physical multi-morbidity used the Internet. Of these, about half (44.6%) used daily, and only 1.2% (63) of users used once a month. The HIT use of the people with multi-morbidity was generally consistent with all respondents, with the majority (38.9%) seeking health information and between 9.5% and 13.8% of Internet users also engaging in the other 3 types of HIT use.

**Table 2:** Frequency of internet and health information technology use among respondents and people with multi-morbidity

Variables	**All respondents (*n* = 7060)**		**Multi-morbidity(*n* = 5380)**	
	n	%	n	%
**Internet use**				
Yes	4234	60.0	3179	59.1
No	2826	40.0	2201	40.9
**Frequency**				
Never	2826	40.0	2201	40.9
Once every few months	199	2.8	166	3.1
About once a month	74	1.0	63	1.2
Several times a month	230	3.3	166	3.1
Several times a week	489	6.9	382	7.1
Everyday	3242	45.9	2402	44.6
**HIT**^**a**^** use in the past 12 months (Yes)**				
Looked up health information on Internet	2749	38.9	2092	38.9
Filled a prescription on Internet	711	10.1	597	11.1
Scheduled medical appointment on Internet	649	9.2	513	9.5
Communicated with health care provider by email	924	13.1	741	13.8

^a^ *HIT* health information technology

Factors that affect Internet and HIT use among people with multi-morbidity were identified in this study. In controlling for other factors, the multivariate logistic regression output shown in Table 3 revealed that female, living in the west area, non-hispanic white, higher education (Some college, College, and Master degree and above), higher family income (200%-399% and ≥ 400%) were significant factors that affect the use of Internet. Older people with advanced age (85 + group: aOR = 0.18, CI = 0.15–0.23, *P* < 0.001) or widowed patients (aOR = 0.66, CI = 0.56–079, *P* < 0.001) were less likely to use the Internet. Among factors such as health status and health service utilization, respondents with poor health status (aOR = 0.53, CI = 0.40–0.70, *P* < 0.001), those without private health insurance (aOR = 0.79, CI = 0.69–0.92, *P* = 0.002) and those who had not talked to a medical specialist in the past year (aOR = 0.79, CI = 0.69–0.91, *P* = 0.001) were less likely to use the Internet. Those without Medicaid (aOR = 1.87, CI = 1.43–2.44, *P* < 0.001), not received home care from health professional (aOR = 1.73, CI = 1.37–2.18, *P* < 0.001), and those with low satisfaction with health care (aOR = 1.61, CI = 1.10–2.36, *P* = 0.014) were more likely to use the Internet.

**Table 3:** Factors influencing internet and HIT use among people with multi-morbidity, results of bivariate and multivariate logistic regression (*n* = 5380)

Variables	Internet use				Any HIT use			
	**uOR **^**a**^** (95%CI**^**c**^**)**	**P-value**	**aOR **^**b**^** (95%CI)**	***P*-value**	**uOR (95%CI)**	***P*-value**	**aOR (95%CI)**	***P*-value**
**Gender**								
Male	1(ref)		1(ref)		1(ref)		1(ref)	
Female	0.83(0.74–0.92)	0.001	1.41(1.22–1.63)	0.000	0.88(0.79–0.98)	0.018	1.36(1.19–1.56)	0.000
**Age**								
65–74	1(ref)		1(ref)		1(ref)		1(ref)	
75–84	0.43(0.38–0.48)	0.000	0.41(0.35–0.48)	0.000	0.48(0.43–0.55)	0.000	0.49(0.42–0.56)	0.000
85 +	0.17(0.14–0.20)	0.000	0.18(0.15–0.23)	0.000	0.18(0.14–0.22)	0.000	0.21(0.16–0.26)	0.000
**Marital Status**								
Currently married	1(ref)		1(ref)		1(ref)		1(ref)	
Widowed	0.36(0.31–0.41)	0.000	0.66(0.56–0.79)	0.000	0.42(0.37–0.48)	0.000	0.75(0.64–0.89)	0.001
Divorced/separated	0.78(0.67–0.91)	0.001	1.05(0.86–1.27)	0.646	0.82(0.71–0.95)	0.000	1.01(0.85–1.21)	0.877
Never married	0.73(0.58–0.93)	0.009	0.87(0.65–1.16)	0.326	0.76(0.60–0.96)	0.009	0.83(0.63–1.08)	0.165
**Region**						0.021		
Northeast	1(ref)		1(ref)		1(ref)	0.003	1(ref)	
Midwest	1.03(0.87–1.23)	0.696	1.03(0.84–1.27)	0.763	0.88(0.75–1.05)	0.153	0.87(0.71–1.06)	0.161
South	0.94(0.81–1.10)	0.462	1.03(0.84–1.25)	0.805	0.89(0.76–1.04)	0.126	0.92(0.76–1.11)	0.368
West	1.29(1.08–1.53)	0.005	1.31(1.05–1.64)	0.016	1.14(0.96–1.35)	0.146	1.11(0.90–1.36)	0.328
**Race**						0.000		
Hispanic	1(ref)		1(ref)		1(ref)	0.000	1(ref)	
Non-Hispanic White	3.17(2.55–3.95)	0.000	2.07(1.58–2.72)	0.000	2.75(2.16–3.50)	0.147	1.72(1.30–2.28)	0.000
Non-Hispanic Black	1.28(0.98–1.67)	0.073	1.11(0.80–1.53)	0.546	1.24(0.93–1.67)	0.001	1.06(0.75–1.49)	0.744
Non-Hispanic Asian	2.18(1.54–3.09)	0.000	1.05(0.68–1.62)	0.816	1.91(1.32–2.76)	0.191	0.95(0.62–1.46)	0.814
Non-Hispanic All other race group	1.43(0.84–2.44)	0.185	1.01(0.55–1.88)	0.966	1.46(0.83–2.59)	0.000	1.10(0.58–2.07)	0.777
**Highest education**						0.000		
High school and below	1(ref)		1(ref)		1(ref)	0.000	1(ref)	
Some college	4.18(3.55–4.91)	0.000	3.13(2.61–3.74)	0.000	3.86(3.29–4.52)	0.000	2.88(2.42–3.42)	0.000
College	4.43(3.62–5.41)	0.000	3.02(2.42–3.77)	0.000	4.33(3.57–5.25)	0.000	3.05(2.48–3.75)	0.000
Master degree and above	9.41(7.98–11.08)	0.000	6.02(4.98–7.28)	0.000	7.46(6.44–8.63)	0.000	4.94(4.18–5.84)	0.000
**Family income to the FPG **^**d**^						0.000		
< 200% FPG	1(ref)		1(ref)		1(ref)	0.000	1(ref)	
200%-399% FPG	2.31(2.03–2.64)	0.000	1.40(1.19–1.64)	0.000	2.18(1.90–2.51)	0.000	1.38(1.17–1.63)	0.000
≥ 400% FPG	6.76(5.77–7.92)	0.000	2.45(2.01–2.99)	0.000	4.93(4.25–5.73)	0.000	1.96(1.62–2.37)	0.000
**Health status**						0.000		
Good	1(ref)		1(ref)		1(ref)		1(ref)	
Fair	0.39(0.34–0.45)	0.000	0.57(0.48–0.68)	0.000	0.51(0.44–0.59)	0.000	0.71(0.59–0.84)	0.000
Poor	0.27(0.22–0.34)	0.000	0.53(0.40–0.70)	0.000	0.35(0.27–0.45)	0.700	0.59(0.43–0.79)	0.001
**Health insurance**						0.000		
Private health insurance(no)	0.57(0.51–0.64)	0.000	0.79(0.69–0.92)	0.002	0.67(0.60–0.75)	0.081	0.93(0.81–1.07)	0.297
Medicare(no)	1.12(0.93–1.34)	0.241	0.83(0.66–1.04)	0.109	1.04(0.87–1.25)	0.000	0.84(0.68–1.05)	0.119
Medicaid(no)	4.47(3.62–5.53)	0.000	1.87(1.43–2.44)	0.000	4.09(3.19–5.23)	0.434	1.95(1.46–2.61)	0.000
Military healthcare insurance(no)	0.92(0.761–1.11)	0.372	0.97(0.77–1.22)	0.785	0.96(0.80–1.16)	0.704	1.04(0.83–1.29)	0.762
**Health care use in the past 12 months**						0.970		
Seen/talked to a medical specialist(no)	0.69(0.62–0.77)	0.000	0.79(0.69–0.91)	0.001	0.55(0.50–0.62)	0.550	0.62(0.54–0.71)	0.000
Seen/talked to a general doctor(no)	0.90(0.76–1.08)	0.262	0.90(0.72–1.11)	0.325	0.85(0.71–1.02)	0.314	0.93(0.76–1.15)	0.512
Received home care from health professional(no)	2.64(2.21–3.15)	0.000	1.73(1.37–2.18)	0.000	1.91(1.59–2.31)	0.000	1.40(1.11–1.76)	0.005
receive care 10 + times(no)	1.29(1.134–1.46)	0.000	1.04(0.88–1.24)	0.652	0.95(0.84–1.08)	0.000	0.83(0.70–0.98)	0.024
**Satisfied with health care**								
Very satisfied	1(ref)		1(ref)		1(ref)		1(ref)	
Somewhat satisfied	0.91(0.80–1.04)	0.165	1.15(0.98–1.36)	0.095	1.00(0.88–1.15)	0.003	1.23(1.05–1.44)	0.009
Somewhat dissatisfied	1.01(0.74–1.38)	0.958	1.61(1.10–2.36)	0.014	1.10(0.81–1.50)	0.153	1.56(1.09–2.25)	0.016
Very dissatisfied	0.72(0.45–1.15)	0.165	1.55(0.87–2.75)	0.139	0.78(0.48–1.27)	0.126	1.43(0.81–2.52)	0.223

^a^ *uOR* Unadjusted odds ratio

^b^ *aOR* adjusted odds ratio

^c^ *CI* Confidence interval

^d^ *FPG* federal poverty guidelines

In the analysis of factors influencing any HIT use in people with multi-morbidity, it was found that female, non-hispanic white, higher educational (Some college, College, and Master degree and above), and higher family income (200%-399% and ≥ 400%) were significant factors influencing any HIT use. Older adults (85 + group: aOR = 0.21, CI = 0.16–0.26, *P* < 0.001) or widowed patients (aOR = 0.75, CI = 0.64–089, *P* = 0.001) were less likely to use any HIT. Among the factors of health status and health care utilization, respondents with poor health status (aOR = 0.59, CI = 0.43 -0.79, *P* = 0.001), those who had not talked to a medical specialist in the past year (aOR = 0.62, CI = 0.54–0.71, *P* = 0.001) and those who had not received care 10 + times in the past year (aOR = 0.83, CI = 0.70–0.98, *P* = 0.024) were less likely to use HIT. Those without Medicaid (aOR = 1.95, CI = 1.46–2.61, *P* < 0.001), not received home care from a health professional in the past year (aOR = 1.40, CI = 1.11–1.76, *P* = 0.005), and with low satisfaction with health care (aOR = 1.56, CI = 1.09–2.25, *P* = 0.016) were more likely to use HIT.

## Discussion

To our knowledge, this is the most recent study using nationally representative data to report on factors associated with Internet use and HIT use affecting older adults with physical multi-morbidity in the U.S. We found that physical multi-morbidity is common among people aged 65 years and older (76.2%). At the same time, older adults are increasingly interested in health information, and the Internet has become an important source in recent years [24]. 60% of the older adults use the Internet, and 38.9% of them rely on it for health information, similar to a previous study [25, 26]. Among older Internet users, 45% of them use the Internet regularly every day. Seeking health information on the Internet was found to be the most frequently reported HIT use behavior among older adults. Following that, communicating with health care providers by email, filling a prescription on the Internet, and scheduling medical appointments on the Internet also accounted for a percentage of Internet use among older Americans. The increasing popularity of HIT among the older adults may, on the one hand, be a natural consequence of information technology developments, given the increasing popularity of the Internet and the ease of use of android, IOS, and other handheld devices [24]. On the other hand, we consider that some older adults with multi-morbidity have limited physical conditions, so online consultation is a more convenient way.

For people with multi-morbidity, the prevalence of their Internet and HIT use varied across sociodemographic factors. Specifically, females were significantly more likely to report using the Internet and HIT. The reasons for this gender difference are unclear, but similar gender-related internet use patterns have been documented in US cancer survivors [27, 28]. The reason may be that the different the role taken up by women in society and the different stressors they face make them more active in obtaining information and seeking social support than men [29], who tend to be more passive information gatherers than active information seekers [30]. Therefore, these differences should be taken into account when designing interventions for Internet and HIT use among people with multi-morbidity. In terms of race, non-Hispanic whites have always been the predominant group using the Internet and HIT. Minority groups such as non-Hispanic Blacks and Hispanics have low participation in HIT, likely due to language and cultural barriers. Also, racial/ethnic minorities have been reported to have more chronic health problems than non-Hispanic whites, which may be a barrier to HIT use [31, 32].

In addition, this study found a strong association between education level and family income on the Internet and HIT use, which is consistent with a Norwegian study that analyzed e-health service use among patients with type 1 and type 2 diabetes and found a positive correlation between education level and search engine use [19]. This may be due to the preferential access and awareness of the Internet, good thinking and economic level of people with higher education levels and household incomes, which provide them with a larger and more comprehensive access to the Internet. Finally, age and widowed marital status are hindering factors for Internet and HIT use. On the one hand, some surveys showed that older adults usually have some degree of cybersecurity concerns and digital skills anxiety [33], further limiting their acceptance and use of new technologies such as the Internet in their sickly physical condition, and on the other hand, widowhood deepens older adults' sense of isolation [34], which may also be detrimental to enhancing their acceptance.

These findings emphasize that health education and interventions to promote the use of the Internet and HIT in older people with multi-morbidity must take into account sociodemographic factors. Older adults with chronic diseases are often unfamiliar with health technologies and may be adversely affected by HIT used in telemedicine and e-health services [35]. Efforts to help them improve their ability to browse and use the Internet and to design age-friendly electronic devices may increase their use of HIT and thus improve their health.

Another key finding of this study is that among factors such as health status and health care utilization, multi-morbidity individuals with poorer health and those who had not talked to a medical professional in the past year were less likely to use the Internet and HIT. This may be because their poor physical condition does not allow them to focus more on new technology and access to new information, and the lack of conversations with doctors may also make them miss the opportunity to be passed on information about HIT use. In addition, we also noticed that patients without Medicaid were more likely to use the Internet and HIT. The American Health Insurance Association describes Medicaid as "a government insurance program for people of all ages with insufficient income and resources to pay for health care [36]." Therefore, this study speculates that this may be a reflection of the fact that people without Medicaid may be financially well off themselves and have more convenient access to the Internet and HIT.

Based on the results of this study, we also found that people with low satisfaction with health care services were more likely to use the Internet and HIT, and this is consistent with the findings of De Rosis S et al. [37]. Patients' low satisfaction with healthcare services is often due to the fact that the healthcare experience did not meet their psychological expectations or that the healthcare services did not adequately address their health problems. As a result, this group of patients may choose the Internet or HIT as a complementary form of service to their consultation. This also suggests that health service providers need to pay more attention to the integration of online and offline healthcare service delivery methods so that patients can be supplemented and re-visited by online treatment in a timely manner when they encounter inadequate offline treatment.

As the Internet plays an increasingly essential role in healthcare, to bridge the digital divide, action is needed to connect and raise awareness among older people, especially those with comorbidities. Hospital systems and providers are increasingly using mobile web-based healthcare services, such as SMS, a popular mobile media platform, as a possible option for health interventions. Mayberry's study of diabetics found that patients with chronic diseases may have easier access to interventions using SMS services than Internet-based platforms, as they use Internet-dependent interventions less than those conducted through SMS text messages [38].

The newly released Healthy People 2030 target revises the key targets for HIT use, the most relevant of which is to increase the proportion of adults who use information technology to track health data or communicate with providers to 87.3% [11]. While these goals reflect the general desire of the general population to increase the use of technology, it remains to be seen whether patients with comorbidities will meet these standards. At the same time, more research on the Internet and HIT use and access among people with chronic diseases is needed to help these populations achieve these goals.

In addition, some previous studies indicated that although many patients have access to the Internet, they may not choose to use HIT resources. This suggests that online forums that disseminate reliable health information and encourage active patient participation can be used to increase patient satisfaction with HIT and increase overall use. The accessibility and convenience of people looking for health information online have increased in the last decade [39, 40], but more research is needed to thoroughly investigate the potential benefits of using HIT in improving the health of older adults. Future research needs to further clarify the information content searched by the older adults on the Internet, such as disease types, treatment strategies, and prescription categories, so as to further information intervention for older patients.

### Limitations

Our study had some limitations. First, the NHIS data were self-reported, and the diagnoses of all chronic diseases may underestimate the true association. Secondly, the NHIS data used in this study is a cross-sectional survey. The study design made it impossible to draw conclusions about possible causal relationships between sociodemographic factors, physical multi-morbidity, and Internet and HIT use. Third, since there are other behavioral factors associated with HIT use that have not been considered, the significant associations observed in the study may be due to unadjusted residual confounders. Finally, the study excluded patients with mental health diseases, which may lead to the limitation of the research conclusions.

## Conclusions

In summary, our study found that Internet and HIT use among older patients with chronic diseases is far from the Healthy People 2030 target. Internet and HIT use vary by some sociodemographic factors. Governments or communities can develop digital skills training and safety awareness campaigns based on the characteristics of geriatric patient networks and HIT use to alleviate geriatric patient skills and safety concerns. Also, medical staff can also encourage older patients to choose a suitable way of using HIT to facilitate their acquisition of health knowledge. It is expected that the use of the Internet and HIT tools will be effective in improving the quality and efficiency of care for patients with chronic or comorbidities, but further research is needed to determine the extent of these health benefits from the trend of increased use of the Internet and HIT.

## Data Availability

All material appearing in this manuscript is in the public domain and may be reproduced or copied without permission. The datasets used and analyzed during the current study are available in https://www.cdc.gov/nchs/nhis/new_nhis.htm.

## Abbreviations

HIT: Health information technology
NHIS: National Health Interview Survey
uOR: Unadjusted odds ratio
aORs: Adjusted Odds Ratios
CI: Confidence Interval
